# Identifying a Role for the Sodium Hydrogen Exchanger Isoform 1 in Idiopathic Pulmonary Fibrosis: A Potential Strategy to Modulate Profibrotic Pathways

**DOI:** 10.3390/biomedicines13040959

**Published:** 2025-04-14

**Authors:** Trina T. Nguyentu, Danielle G. Vigilante, Mishika Manchanda, Meera S. Iyer, Sara Desalegne, Joseph J. Provost

**Affiliations:** 1 Department of Biomedical Engineering, Duke University, Durham, NC 27708, USA; trina.nguyentu@duke.edu; 2 Department of Chemistry and Biochemistry, University of San Diego, San Diego, CA 92110, USA; dvigilante@sandiego.edu (D.G.V.); mmanchanda@sandiego.edu (M.M.); miyer2001@gmail.com (M.S.I.); sdesalegne@sandiego.edu (S.D.)

**Keywords:** NHE1 (sodium hydrogen exchanger isoform 1), lung fibrosis, cytoskeletal remodeling, fibroblast, myofibroblast differentiation, transforming growth factor-beta (TGFβ), lysophosphatidic acid (LPA), serotonin (5HT), interleukin-1 (IL-1), interleukin-6 (IL-6)

## Abstract

**Background/Objectives**: Idiopathic pulmonary fibrosis (IPF) is a chronic lung disease characterized by excessive extracellular matrix (ECM) production and tissue stiffening, resulting in impaired lung function. Sodium hydrogen exchanger isoform 1 (NHE1) is a key mediator of intracellular and extracellular pH regulation, influencing fibroblast activation, motility, and proliferative pathways. This study investigates the role of NHE1 in actin stress fiber formation, fibroblast-to-myofibroblast differentiation, and cytokine secretion in IPF progression. **Methods**: Fibroblasts were treated with profibrotic agonists, including transforming growth factor-beta (TGFβ), lysophosphatidic acid (LPA), and serotonin (THT), in the presence or absence of the NHE1-specific inhibitor, EIPA. Actin stress fibers were visualized using phalloidin staining, while α-smooth muscle actin (α-SMA) expression and cytokine secretion (TGFβ, IL-6, and IL-8) were quantified using immunostaining and ELISA. Intracellular pH changes were measured using BCECF-AM fluorescence. **Results**: Profibrotic agonists induced significant actin stress fiber formation and α-SMA expression in fibroblasts, both of which were abolished by EIPA. NHE1 activity was shown to mediate intracellular alkalization, a critical factor for fibroblast activation. Cytokine secretion, including TGFβ, IL-6, and IL-8, was enhanced by agonist treatments but reduced with NHE1 inhibition. Chronic TGFβ exposure increased intracellular pH and sustained myofibroblast differentiation, which was partially reversed by EIPA. **Conclusions**: NHE1 is indicated to play a novel and potential role in processes supporting profibrotic agonists driving fibroblast activation and IPF progression. Targeting NHE1 could present a potential therapeutic approach to disrupt profibrotic pathways and mitigate IPF severity.

## 1. Introduction

Idiopathic pulmonary fibrosis (IPF) is a chronic, progressive lung disease characterized by dysregulated wound healing that results in the increased production of extracellular matrix (ECM) and scarring tissue in the lung interstitium, ultimately compromising lung structure and function [1]. Under normal conditions, within the lung tissues, fibroblasts are a key cell type responsible for the growth, repair, and health of connective tissue and ECM homeostasis [2,3].

However, in IPF, the activity of fibroblasts is disrupted by increased levels of profibrotic agonists, which drive the migration, proliferation, and differentiation of fibroblasts into active profibrotic myofibroblasts, a hallmark of fibrotic diseases [1,4]. Myofibroblasts are often characterized by the expression of alpha-smooth muscle actin (α-SMA) and the formation of intracellular actin stress fibers, both attributed to the cell type’s highly contractile phenotype. These actin stress fibers are bundles of polymerized protein filaments that stretch throughout the cell, forming contractile actin–myosin complexes that exert stiffness within the ECM [4]. The fibroblast–myofibroblast differentiation is an inflammatory response to injury. Once activated, myofibroblasts also secrete excessive amounts of ECM fibrillar proteins such as collagens and fibronectins throughout the lung interstitium and drive fibrotic tissue remodeling.

This pathological remodeling process, combined with the accumulation of actin stress fibers and increased ECM production, not only stiffens lung tissue but also disrupts alveolar architecture. As a result, the affected patient’s alveolar architecture becomes distorted, leading to lung inflexibility, impaired gas exchange, respiratory failure, and ultimately death. IPF has an unknown etiology and limited treatment options. As a result, the prognosis for IPF patients is quite poor, with a median survival of just 3–5 years if left untreated [5].

Tissue stiffening significantly influences fibrosis progression by activating transforming growth factor-β (TGFβ), a profibrotic cytokine involved in wound healing and tissue repair. Under normal physiological conditions, TGFβ initiates a cascade of intracellular signaling pathways involved in cell proliferation, migration, differentiation, and ECM remodeling [6,7]. Latent TGFβ is sequestered in the ECM as part of a large latent complex, preventing its premature activation and signaling. Extracellular acidification activates TGFβ by altering the activity of ECM-associated proteases, such as matrix metalloproteinases (MMPs) and plasmin, which cleave the latent complex and release the mature, active ligand. Acidification can also disrupt interactions between latent TGFβ-binding proteins (LTBPs) and the ECM, facilitating proteolytic cleavage and exposure of the receptor-binding domains of TGFβ, thereby enhancing its bioavailability and downstream signaling [8].

Other factors involved in fibrosis progression include profibrotic hormones and cytokines such as lysophosphatidic acid (LPA), serotonin (5-hydroxytryptamine, 5HT), interleukin-1β (IL-1β), and interleukin-6 (IL-6). These molecules play overlapping roles in underlying fibrotic tissue remodeling. LPA mediates cytoskeletal reorganization and cell motility in degenerative processes, leading to stress fiber formation and enhanced fibroblast migration and recruitment [9,10]. 5HT promotes cell migration, proliferation, and cytokine production, facilitating the formation of temporary scar tissue for tissue regeneration [11]. However, in chronic injury, 5HT signaling promotes aberrant wound healing, resulting in tissue fibrosis and impaired organ regeneration [12]. IL-1β is an inducible cytokine essential for host response and epithelial wound repair. In fibrotic conditions, it exacerbates tissue damage during chronic disease and acute injury, promoting an inflammatory environment [13,14]. IL-6 mediates inflammation and repair and is involved in wound healing [15]. However, in fibrotic conditions, IL-6 promotes trans-signaling in lung fibroblasts and other cells to promote ECM production, cell proliferation, migration, and sustained inflammation, which increase the fibrotic response [13,15]. Collectively, the interplay and persistent activities of these factors contribute to the complex and dynamic environment that promotes profibrotic pathways in fibrosis.

Fibrosis progression is driven by a variety of factors, including signaling cascades, cellular responses, and the surrounding microenvironment. A key aspect to these processes is the regulation of intracellular (pHi) and extracellular pH (pHe) in maintaining cellular homeostasis. The control of this pH gradient across a membrane supports the increase of cell proliferation, the activation of extracellular proteases, and enhanced motility—a process that influences cell behavior and matrix modeling [16]. The sodium hydrogen exchanger isoform 1 (NHE1) is an ion transporter protein ubiquitously found in the plasma membrane that regulates pHi and pHe. Structurally, NHE1 has 12 transmembrane segments with an N-terminus transporter domain and an extended C-terminal regulatory domain. Together, these domains facilitate the exchange of intracellular H^+^ ions for extracellular Na^+^ ions, maintaining pH homeostasis. In our study, we focus on CCL39 fibroblasts which have long been used to study NHE1 as it is the only isoform expressed in these cells, unlike other fibroblasts [17,18]. NHE1 has been implicated in fibrotic diseases and cancer due to its role in ion transport, cytoskeletal organization, and signaling [16].

In cancer and tumor microenvironments, NHE1 activity is elevated, leading to alkaline pHi and acidic pHe. This dysregulated pH environment supports tumor growth, motility invasion, and metastasis and facilitates ECM breakdown [19]. Beyond its role as an ion transporter protein, NHE1 also acts as a scaffold for protein signaling complexes due to its C-terminal regulatory domain acting as a docking site for this assembly. This scaffolding function provides a structural anchor of the cytoskeletal proteins to the plasma membrane, which contributes to matrix remodeling, cell migration through the formation of cellular protrusions, such as lamellipodia and invadopodia, and pH homeostasis—central processes to both cancer metastasis and fibrosis progression [16,19].

Interestingly, several signaling pathways activated by NHE1 in cancer cells are also implicated in profibrotic behaviors observed in fibroblasts and myofibroblasts. For instance, pathways promoting cytoskeletal reorganization and ECM remodeling in cancer are similar to fibrotic tissues, highlighting the shared mechanisms between cancer and IPF progression [20]. Although the factors supporting IPF advancement remain unclear, the parallels between IPF and cancer provide insight into the role of NHE1 as a key mediator in fibrotic diseases.

Exploring the effects of classical agonists such as TGFβ, 5HT, and LPA can provide valuable information about the underlying mechanisms of IPF and potentially lead to better treatments. Because NHE1 plays a critical role in ECM remodeling, cell growth, and motility, we hypothesize that NHE1 supports fibrosis progression by facilitating actin stress fiber formation, fibroblast-to-myofibroblast differentiation, and cytokine secretion. Lung fibroblasts treated with profibrotic agonists like TGFβ, 5HT, and LPA show stress fiber formation and α-SMA expression, which are suppressed by the NHE1 inhibitor EIPA. Additionally, IL-6 secretion and TGFβ expression induced by LPA, 5HT, and IL-1β are blocked by NHE1 inhibition. These findings begin to establish NHE1 as essential for fibroblast activation and profibrotic signaling, highlighting its potential as a therapeutic target in IPF.

## 2. Materials and Methods

### 2.1. Cell Culture

CCL39 and WI38 fibroblasts were cultured in high-glucose DMEM supplemented with 10% fetal bovine serum (FBS), 100 U/mL penicillin, and 100 µg/mL streptomycin at 37 °C in a 5% CO_2_ atmosphere. Cells and all culture materials were purchased from ATCC, Manassas, VA, USA. Cells were passaged at 70–80% confluence and maintained in growth media unless otherwise specified. All peptide agonists (TGFβ, ILI1, ILI6, and ILI8) were purchased from R&D Systems as recombinant active form peptides. Phenylephrine hydrochloride (PE) and 5HT were purchased from Fisher Scientific (Hampton, NH, USA).

### 2.2. Actin Stress Fiber Staining and Quantification

F-actin formation was determined in CCL39 cells after seeding on sterile glass coverslips and grown to approximately 50% confluence. For stress fiber visualization, cells were fixed in 3% paraformaldehyde for 30 min at 4 °C, permeabilized with 0.4% Triton X-100 for 10 min, and incubated with Alexa Fluor 488-conjugated phalloidin (0.5 µg/mL, Fisher Scientific USA) for 60 min. Coverslips were mounted using Prolong Antifade Reagent (Invitrogen, Waltham, MA, USA). Fluorescence images were captured with an Evos inverted microscope (Invitrogen USA), and stress fiber-containing cells were counted in five random fields per slide. Percentages of stress fiber-positive cells were calculated as a proportion of total cells in the field.

α-Smooth Muscle Actin (α-SMA, Fisher Scientific USA) Staining: After fixing and permeabilizing as described above, cells were incubated with a red fluorophore-conjugated α-SMA antibody (1:200 dilution) in PBS with 1% BSA overnight at 4 °C. After three PBS washes, nuclei were counterstained with DAPI (1 µg/mL, Invitrogen USA), and coverslips were mounted as above. Fluorescent images were collected using a Evos inverted microscope using a 20× objective to assess α-SMA expression and localization.

### 2.3. Intracellular pH Determination

Cells were seeded onto opaic black 96-well plates and incubated with 3 µM BCECF-AM (Invitrogen USA) in HEPES-buffered saline (145 mM NaCl, 3 mM KCl, 1 mM MgCl_2_, 1 mM CaCl_2_, and 5 mM glucose; pH 7.4) for 20 min at 37 °C. For pH calibration, cells were equilibrated with K^+^-nigericin buffers of known pH. BCECF fluorescence was excited alternately at 502 nm and 439 nm, and emission at 525 nm was measured using a fluorescent plate reader. Intracellular pH was calculated from fluorescence ratios based on a standard curve.

### 2.4. ELISA for Cytokine Quantification

Supernatants (100 µL) from cells cultured in 6-well plates were collected and processed using an ELISA assay (Human TGFβ1, IL6, and ILI8 ELISA Kits, RayBio Fisher Scientific, Corners, GA, USA) according to the manufacturer’s instructions. The cells were treated with the indicated agonist for 12 h prior to collection. Samples and standards were incubated in pre-coated 96-well plates, followed by sequential washes and incubation with biotinylated antibodies, HRP-streptavidin, and TMB substrate. Absorbance at 450 nm was measured using a microplate reader (Agilent, Highland Park, VT, USA). Cytokine concentrations were calculated using a log-log standard curve and normalized within each group.

## 3. Results

### 3.1. NHE1 Activity Is Essential for Agonist-Induced Stress Fiber Formation in CCL39 Cells

To investigate the role of NHE1 in stress fiber formation, CCL39 lung fibroblasts, chosen for their exclusive expression of NHE1, were serum-starved (0.1% FBS) for 12–14 h before stimulation with TGFβ. The cells were pretreated with 5-(*N*-ethyl-*N*-isopropyl) amiloride (EIPA; 10 µM), a specific NHE1 inhibitor, 15 min prior to TGFβ stimulation to evaluate the effect of NHE1 inhibition. Following treatment, the cells were stained with Alexa Fluor 488-conjugated phalloidin to visualize F-actin stress fibers [21].

**Figure 1:**
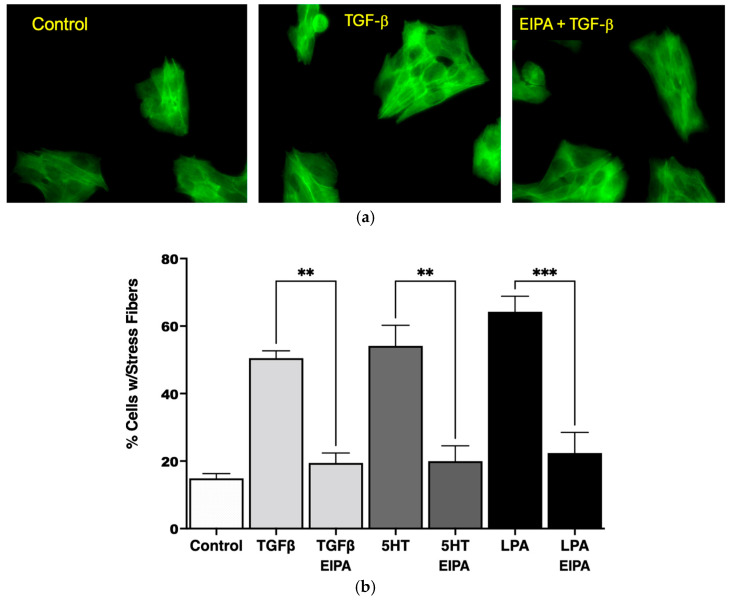
NHE1 activity is required for stress fiber formation in CCL39 cells in response to profibrotic agonists. (**a**) Representative fluorescence microscopy images at 20X magnification of CCL39 fibroblasts stained with Alexa Fluor 488-conjugated phalloidin to visualize F-actin stress fibers (green). Control cells (serum-starved, 0.1% FBS) exhibit diffuse actin distribution with no organized stress fibers. Cells treated with TGFβ (2 ng/mL for 15 min) display strong actin stress fibers spanning the cytoplasm and crossing through the nucleus. Pretreatment with the NHE1-specific inhibitor 5-(*N*-ethyl-*N*-isopropyl) amiloride (EIPA, 10 µM) or vehicle prior to TGFβ stimulation abolishes stress fiber formation, resulting in uniform actin distribution throughout the cytoplasm. (**b**) Quantification of the percentage of cells with stress fibers under different treatment conditions. Serum-starved control cells show minimal stress fiber formation (14% ± 1.3%). Treatment with TGFβ (2 ng/mL), 5HT (10 µM), or LPA (100 nM) significantly increases the percentage of cells displaying stress fibers to 53% ± 3.1%, 57% ± 6.3%, and 63% ± 4.2%, respectively. Pretreatment with EIPA significantly reduces stress fiber formation in all agonist-treated groups, restoring percentages to levels comparable to control cells. Data are presented as mean ± SEM, with statistical significance using a two-way ANOVA to assess the main effects of agonist stimulation and inhibitor treatment. Post hoc comparisons were conducted using Tukey’s test. ** *p* < 0.01 for TGFβ and 5HT, and *** *p* < 0.001 for LPA (n = 5 independent experiments).

As shown in Figure 1a, the serum-starved control cells lacked organized stress fibers, with actin filaments distributed diffusely throughout the cytoplasm. In contrast, treatment with TGFβ (2 ng/mL for 15 min) induced the formation of robust stress fibers spanning across the cytoplasm and through the nucleus. However, pretreatment with EIPA prior to TGFβ exposure prevented the formation of stress fibers, leaving actin filaments evenly dispersed throughout the cytoplasm. These results demonstrate that TGFβ-induced stress fiber formation is dependent on NHE1 activity.

The quantification of stress fiber formation under different agonist treatments is presented in Figure 1b. TGFβ treatment increased the percentage of cells with stress fibers significantly (53% ± 3.1%) compared to untreated control cells (14% ± 1.3%). Similarly, treatment with 5HT (10 µM) and LPA (100 nM) led to robust stress fiber formation, with 57% ± 6.3% and 63% ± 4.2% of cells forming stress fibers, respectively. Notably, the induction of stress fibers by these agonists was completely abolished in the presence of EIPA, with the percentage of cells forming stress fibers returning to control levels for TGFβ (** *p* < 0.01), 5HT (** *p* < 0.01), and LPA (*** *p* < 0.001).

This aligns with previous studies showing that TGFβ promotes stress fiber formation and myofibroblast differentiation in lung fibroblasts. For instance, TGFβ1-induced stress fiber assembly has been documented in MRC-5 lung fibroblasts, where it contributes to increased contractility and extracellular matrix production, both hallmark features of pulmonary fibrosis [22,23]. Similarly, serotonin (5HT) has been implicated in fibrotic processes by enhancing fibroblast activation and promoting cytoskeletal remodeling, partly through its ability to modulate TGFβ production [24]. This suggests a synergistic relationship between 5HT and TGFβ signaling in the induction of stress fiber formation. Additionally, LPA, a known activator of cytoskeletal reorganization, has previously been shown to induce stress fibers in fibroblasts, consistent with the observations in this study [25].

These findings collectively demonstrate that NHE1 activity is critical for the cytoskeletal remodeling induced by profibrotic agonists such as TGFβ, 5HT, and LPA. The dependency of stress fiber formation on NHE1 activity underscores its role in the mechanostiffening of fibroblasts, a key feature of idiopathic pulmonary fibrosis (IPF) progression.

### 3.2. Agonist-Induced Alkalization Confirms NHE1 Activity in CCL39 Cells

To evaluate whether the profibrotic agonists that promote stress fiber formation also stimulate NHE1 activity, intracellular pH (pHi) was measured before and after treatment with TGFβ, 5HT, or LPA. The resting pHi of control serum-starved CCL39 cells ranged between 7.1 and 7.2. Treatment with each agonist resulted in the significant alkalization of pHi, indicative of increased NHE1-mediated proton transport.

**Figure 2:**
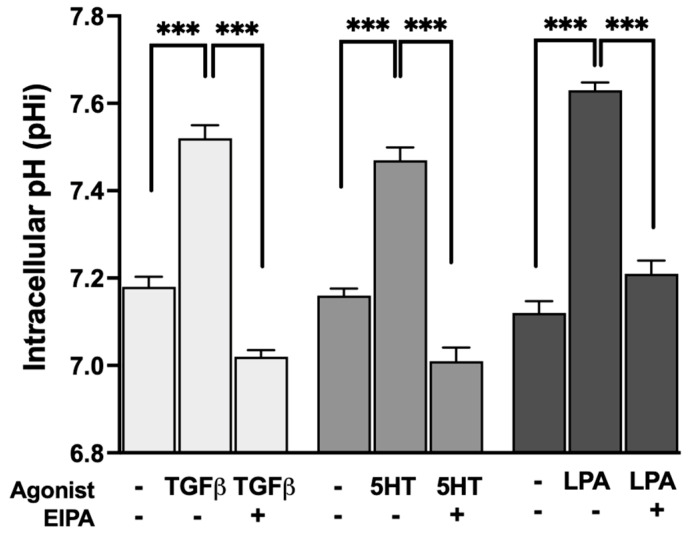
Agonist-induced stimulation of NHE1 activity measured by intracellular pH (pHi) alkalization in CCL39 cells. CCL39 fibroblasts were treated with TGFβ (2 ng/mL), 5HT (10 µM), or LPA (100 nM), and intracellular pH (pHi) was measured before and after treatment to assess NHE1-mediated proton transport. Resting pHi in control cells ranged from 7.1 to 7.2. Treatment with TGFβ, 5HT, and LPA resulted in the significant alkalization of pHi, indicating increased NHE1 transport activity. Post-treatment pHi values were 7.52 ± 0.03 for TGFβ, 7.47 ± 0.029 for 5HT, and 7.63 ± 0.03 for LPA, demonstrating that these agonists stimulate NHE1 activity. Each agonist also aligns with their requirement for NHE1 activity in promoting F-actin stress fiber formation. Data represent the mean ± SEM from n = 5 independent experiments. A two-way ANOVA was performed to assess the main effects of agonist stimulation and inhibitor treatment. Post hoc comparisons were conducted using Tukey’s test. Significant differences: *p* < 0.05 compared to control, agonist alone, or inhibitor alone. Statistical significance annotations are indicated on the graph (*** *p* < 0.001).

As shown in Figure 2, TGFβ treatment increased pHi to 7.52 ± 0.03, while 5HT and LPA raised pHi to 7.47 ± 0.029 and 7.63 ± 0.03, respectively (n = 5). These results confirm that all three agonists stimulate NHE1 activity, consistent with their dependence on NHE1 for inducing F-actin stress fiber formation. The observed pHi alkalization reflects the enhanced exchange of intracellular H^+^ ions for extracellular Na^+^ ions, a hallmark of NHE1 activation.

These results are consistent with previous studies showing that TGFβ [26,27] and 5HT [28] increase NHE1 activity, likely through pathways that coordinate intracellular pH regulation with cytoskeletal dynamics and contractility. In addition to its role in cytoskeletal remodeling, NHE1 activity also modulates TGFβ-driven cellular proliferation and differentiation. Previous studies have demonstrated that NHE1 enhances TGFβ signaling by increasing intracellular pH, which promotes TGFβ receptor activity and downstream Smad phosphorylation. This amplification of TGFβ signaling is crucial for fibroblast proliferation and the transition to a myofibroblast phenotype. Our findings of TGFβ-induced pHi alkalization align with these observations, suggesting that NHE1 contributes to both the structural and functional aspects of fibroblast activation during fibrosis [27].

Similarly, 5HT and LPA, which are known to regulate cytoskeletal reorganization through NHE1, may also influence cellular proliferation via their effects on intracellular pH. Together, these results highlight NHE1 as a central mediator of the fibroblast response to profibrotic agonists, linking its activity to both structural cytoskeletal changes and functional behaviors such as proliferation and differentiation.

### 3.3. NHE1 Activity Is Required for TGFβ-Induced α-Smooth Muscle Actin (α-SMA) Expression

**Figure 3:**
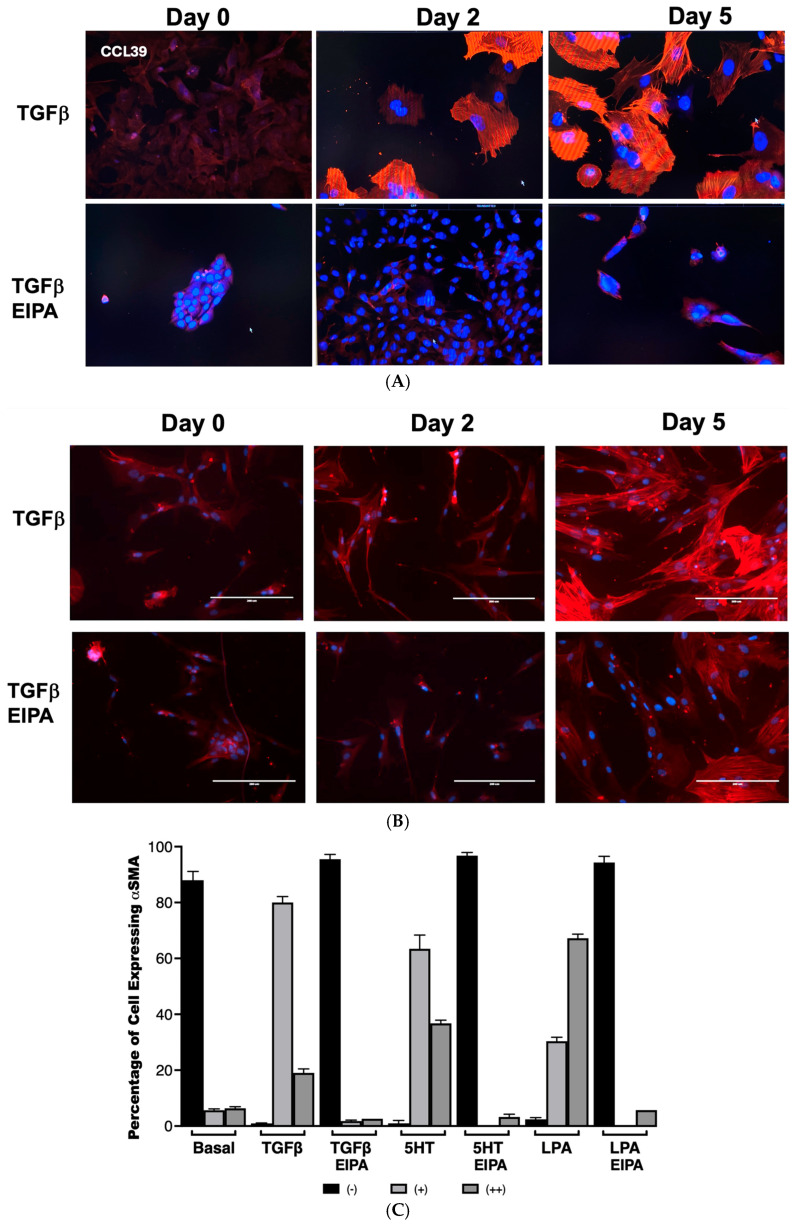
NHE1 activity is critical for TGFβ-induced myofibroblast differentiation and α-SMA expression. (**A**) Representative images of CCL39 cells treated with TGFβ (2 ng/mL) for 0, 2, and 5 days, stained for α-smooth muscle actin (α-SMA) to visualize myofibroblast differentiation taken with a 20 X magnification. At day 2, most cells displayed robust α-SMA expression, which became more widespread and intense by day 5. Similar results were observed in non-fibrotic WI38 cells treated with TGFβ for 5 days, confirming the induction of myofibroblast differentiation. Co-treatment with EIPA (10 µM), an NHE1-specific inhibitor, blocked α-SMA expression in both cell types, demonstrating that NHE1 activity is required for TGFβ-induced transformation. (**B**) Representative images of WI38 cells treated with TGFβ, 5HT (10 µM), or LPA (100 nM), showing α-SMA expression at day 2. All agonists robustly induced α-SMA expression, while co-treatment with EIPA significantly reduced or eliminated detectable α-SMA expression. (**C**) Quantification of α-SMA expression in CCL39 cells treated with TGFβ, 5HT, or LPA for 2 days, with and without EIPA co-treatment. Cells were categorized as displaying no detectable α-SMA (−), moderate α-SMA expression (+), or intense α-SMA expression (++). In control cells, 88% ± 2.12% exhibited no α-SMA expression. TGFβ induced α-SMA in 99% of cells (80% ± 2.11% with moderate expression and 19% ± 1.4% with intense expression). Similarly, 5HT and LPA treatments resulted in α-SMA expression in 100% of cells, with strong expression (++): 36.7% ± 1.14% for 5HT and 67.2% ± 1.45% for LPA. Co-treatment with EIPA reduced α-SMA expression to undetectable levels (94–96% of cells with no detectable α-SMA). Data represent the mean ± SEM from n = 4 independent experiments.

To examine the role of NHE1 in myofibroblast differentiation, the expression of α-smooth muscle actin (α-SMA), a hallmark of myofibroblast activation, was assessed in CCL39 cells treated with TGFβ over 0, 2, and 5 days (Figure 3A). On day 2, nearly all cells displayed robust α-SMA expression, which was sustained and intensified by day 5, demonstrating the ability of TGFβ to effectively transform fibroblasts into myofibroblasts. To account for other NHE isoforms and emphasize that these processes happen in human cells, we also examined WI38 non-fibrotic human fibroblasts treated with TGFβ (2 ng/mL) for 2 and 5 days. In these cells, α-SMA staining was similarly prominent, indicating that TGFβ activates fibrotic processes in both cell lines. Notably, co-treatment with EIPA, an NHE1-specific inhibitor, blocked most of the α-SMA expression in both cell types (with some WI38 cells still displaying α-SMA), highlighting the critical role of NHE1 activity in facilitating this transformation (Figure 3B). That α-SMA was expressed in WI38 cells could be due to other pHi-regulating steps including other NHE isoforms.

The quantification of α-SMA expression in CCL39 cells treated with TGFβ, 5HT, or LPA was performed to further analyze the dependence of this fibrotic marker on NHE1 activity (Figure 3B,C). In untreated control cells, α-SMA was undetectable in 88% ± 2.12% of the cell population. However, after two days of TGFβ treatment, 99% of cells expressed α-SMA, with 80% ± 2.11% showing moderate staining (+) and 19% ± 1.4% displaying intense expression (++). Similar patterns were observed with other profibrotic agonists: 5HT induced moderate α-SMA expression in 63.4% ± 4.94% of cells and strong expression in 36.7% ± 1.14%, while LPA-treated cells displayed moderate expression in 30.3% ± 1.45% and strong expression in 67.2% ± 1.45% of cells. In contrast, the presence of EIPA significantly inhibited α-SMA expression, with 94–96% of cells showing no detectable α-SMA across all agonist treatments.

These findings establish that NHE1 activity is essential for the induction of α-SMA by TGFβ, 5HT, and LPA, linking its activity to fibroblast-to-myofibroblast differentiation. While previous studies have demonstrated a role for NHE1 in cytoskeletal remodeling and differentiation in other contexts, such as cardiomyocyte differentiation [29], no prior research has directly connected NHE1 to fibroblast transformation into myofibroblasts in idiopathic pulmonary fibrosis (IPF). This work represents the first demonstration of NHE1’s involvement in this critical fibrotic process, suggesting that NHE1-mediated ion transport and cytoskeletal interactions are central to the progression of IPF.

### 3.4. Basal Intracellular pH Changes After Chronic TGFβ Exposure and Fibroblast-to-Myofibroblast Transition

**Figure 4:**
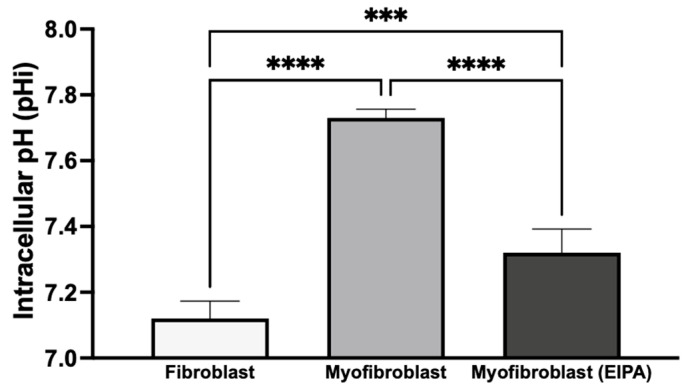
Basal intracellular pH changes after chronic TGFβ exposure and transition from fibroblasts to myofibroblasts. CCL39 fibroblasts were cultured in high-glucose DMEM with 10% FBS in the absence (resting fibroblasts) or presence (myofibroblasts) of 2 ng/mL TGFβ for five days. Cells were subsequently incubated in TGFβ-free medium for 12 h prior to pHi measurement using BCECF-AM. Resting fibroblasts exhibited a basal pHi of 7.12 ± 0.053, while transformed myofibroblasts showed an elevated pHi of 7.73 ± 0.026. The treatment of myofibroblasts with 10 μM EIPA, an NHE1 inhibitor, reduced pHi to 7.32 ± 0.072. Significant differences: *p* < 0.05 compared to control, agonist alone, or inhibitor alone. Statistical significance annotations are indicated on the graph (*** *p* < 0.001 or (**** *p* < 0.0001). The sustained alkaline pHi in myofibroblasts suggests the activation of additional proton transport mechanisms alongside NHE1.

To investigate basal intracellular pH (pHi) and the role of NHE1 following chronic TGFβ exposure, we treated CCL39 fibroblasts in the presence or absence of TGFβ for five days. To determine the impact on cellular transformation and not the growth factor directly, cells were maintained in TGFβ-free medium for 12 h before pHi measurement. As shown in Figure 4, resting fibroblasts exhibited a pHi of 7.12 ± 0.053, while transformed myofibroblasts had a higher pHi of 7.73 ± 0.026, indicating an alkaline shift during the fibroblast-to-myofibroblast transition. To evaluate the contribution of NHE1 to this shift, transformed myofibroblasts were treated with 10 μM EIPA for 30 min before pHi measurement. This treatment reduced the pHi to 7.32 ± 0.072 but did not fully restore it to the level observed in resting fibroblasts. This partial reduction suggests that other proton transporters, such as the H^+^-ATPase, H^+^/K^+^-ATPase, and the monocarboxylate transporters, may also contribute to the elevated pHi in myofibroblasts.

These findings indicate that chronic TGFβ exposure induces a stable transformation of fibroblasts into myofibroblasts, leading to a sustained increase in intracellular pH even after TGFβ withdrawal. The elevated pHi in myofibroblasts may result from the activation of autocrine signaling pathways or the secretion of alternative agonists that regulate NHE1 activity, along with the potential contributions from other proton transport mechanisms. This sustained increase in pHi likely supports the enhanced growth and motility associated with myofibroblast activation.

### 3.5. Agonist-Induced Cytokine Secretion and the Role of NHE1 in CCL39 Cells

To investigate the relationship between cytokine secretion and NHE1 activity in CCL39 cells, we examined the effects of a panel of agonists known for their roles in fibrosis and GPCR-mediated signaling. These included LPA, PE, 5HT, tumor necrosis factor-alpha (TNFα), TGFβ, IL-1, IL-6, and IL-8. These agonists activate pathways implicated in fibrotic development and have been shown to modulate processes like cell growth, motility, and cytokine release, often through mechanisms involving NHE1. To determine the role of NHE1 in cytokine secretion, cells were co-treated with the NHE1 inhibitor EIPA during agonist stimulation.

**Figure 5:**
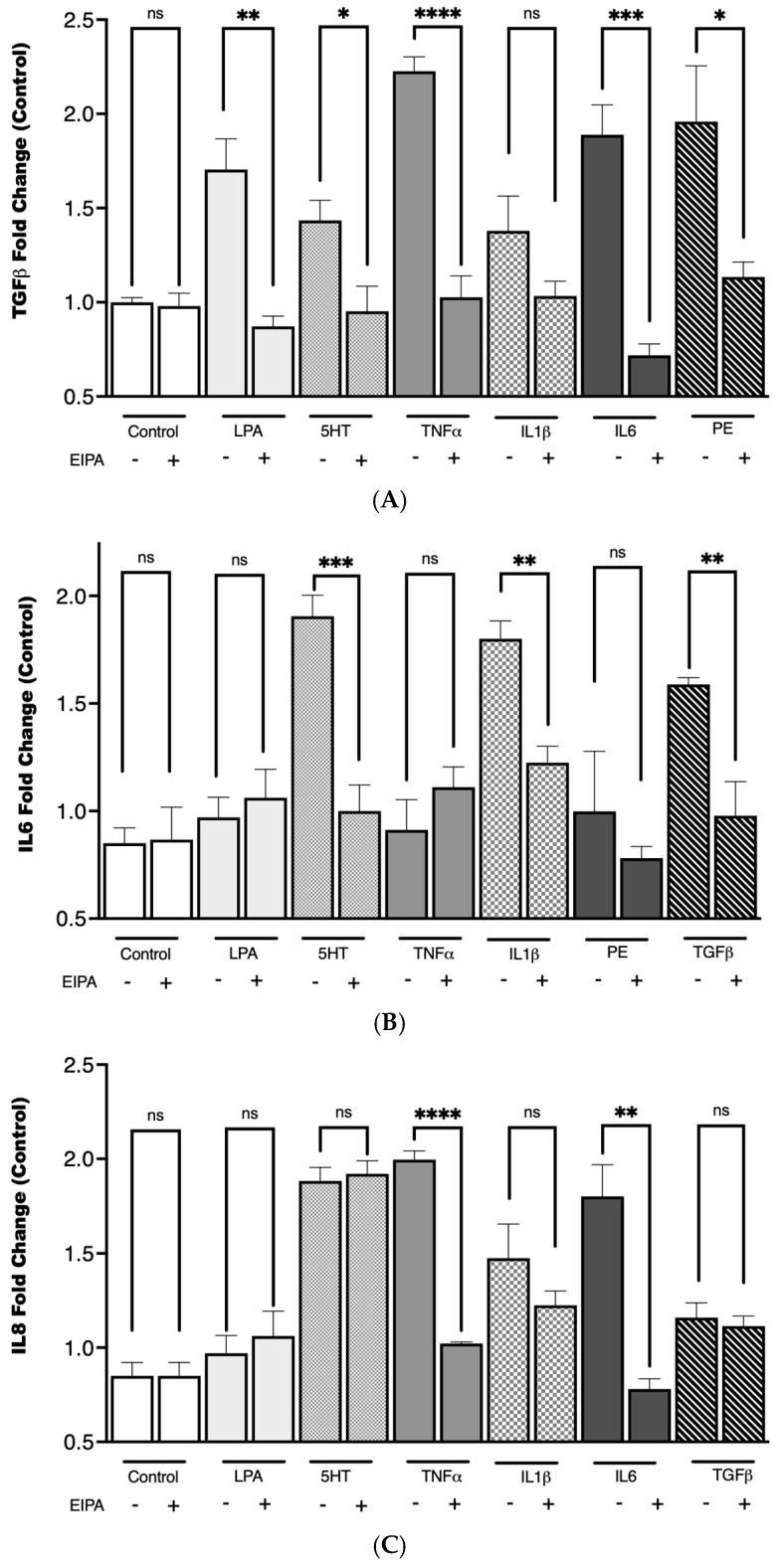
TGFβ secretion following agonist stimulation and NHE1 inhibition. CCL39 cells were stimulated with the indicated agonist to evaluate their impact on cytokine secretion. The CCL39 cells were seeded and incubated until 80% confluence. The medium was removed and replaced with fresh high-glucose DMEM containing reduced serum (0.1% FBS) for 15 min prior to the addition of the agonist. To measure the impact of NHE1, cells were pretreated with 10 μM EIPA in addition to low-serum medium. After 30 min of agonist (or vehicle control) treatment, 100 µL of cell lysate was captured and used to detect TGFβ (**A**), IL6 (**B**), and IL8 (**C**). Data are presented as mean ± SEM (n = 5). A two-way ANOVA was used to evaluate the main effects of agonist stimulation and inhibitor treatment. Tukey’s post hoc test was applied to determine group difference. (ns: not significant, * *p* < 0.1, ** *p* < 0.01, *** *p* < 0.001, **** *p* < 0.0001).

As shown in Figure 5, TGFβ secretion increased with the addition of LPA (** *p* < 0.01), 5HT (* *p* < 0.1), TNFα (**** *p* < 0.0001), IL-6 (*** *p* < 0.001), and PE (**** *p* < 0.0001), while IL1 did not. IL-6 secretion was upregulated only by 5HT (**** *p* < 0.0001), IL1 (*** *p* < 0.001), and TGFβ (*** *p* < 0.001). LPA, phenylephrine, 5HT (*** *p* < 0.001), TNFα (**** *p* < 0.0001), and IL-6 (*p* < 0.01) stimulation significantly increased IL-8 secretion. In each case, except for IL-8 secretion with 5HT, stimulation pretreatment with EIPA reduced cytokine secretion. The increase in IL-6 secretion in response to TGFβ stimulation supports the synergistic relationship between these cytokines in amplifying fibrotic pathways. Notably, IL-1 did not significantly impact TGFβ levels. These findings align with the well-established role of TGFβ in activating fibroblast differentiation and ECM production.

Pretreatment with EIPA significantly reduced cytokine secretion across most conditions, reinforcing the idea that NHE1 activity is integral to the signaling pathways driving cytokine release and the establishment of a pro-fibrotic microenvironment. This could suggest that a distinct but interconnected cytokine network involving TGFβ, IL-6, and IL-8 may rely on NHE1-mediated intracellular pH regulation for their pro-fibrotic effects, with variations in dependence reflecting pathway-specific roles and compensatory mechanisms in fibrosis progression.

The agonists tested in this study not only share roles in fibrosis individually but may also act synergistically or sequentially to sustain the fibrotic environment. For instance, the upregulation of TGFβ secretion by LPA and 5HT could further enhance the activation of fibroblasts into myofibroblasts, creating a feedback loop that amplifies fibrosis. Similarly, IL-6 and IL-8, known to contribute to chronic inflammation, could interact with TGFβ to exacerbate tissue remodeling and extracellular matrix deposition. The inhibition of cytokine secretion by EIPA underscores the importance of NHE1 in these processes, suggesting that targeting NHE1 might be a potential therapeutic approach to disrupt this cascade.

### 3.6. De-Differentiation of Myofibroblasts via NHE1 Inhibition

**Figure 6:**
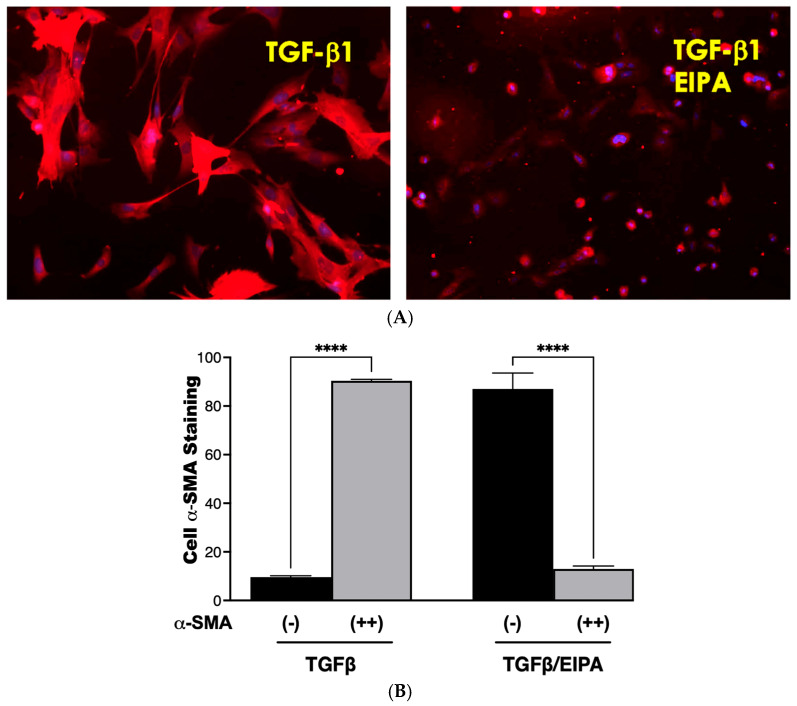
De-differentiation of myofibroblasts. (**A**) Representative images of α-SMA staining in fibroblasts treated with TGFβ for seven days and subsequently cultured for five days in either TGFβ-containing medium or TGFβ with the NHE1 inhibitor, EIPA taken with a 20 X magnification. Cells cultured with TGFβ alone displayed strong α-SMA staining, indicative of myofibroblast differentiation. Co-incubation with EIPA led to a noticeable reduction in α-SMA staining, suggesting the potential de-differentiation of myofibroblasts. (**B**) Quantification of α-SMA staining from three independent trials. Black bars represent the percentage of cells with minimal α-SMA staining, while grey bars indicate cells with moderate-to-strong α-SMA staining. Fibroblasts cultured with TGFβ alone displayed 90.4% ± 0.57% moderate-to-strong α-SMA staining, consistent with sustained myofibroblast differentiation. In contrast, co-incubation with TGFβ and EIPA significantly reduced moderate-to-strong α-SMA staining to 13% ± 1.6% (**** *p* < 0.001), supporting the role of NHE1 inhibition in promoting de-differentiation.

Fibroblasts were activated to myofibroblasts through a 7-day incubation with TGFβ, followed by continued culture for five days in either TGFβ-containing medium or TGFβ combined with the NHE1 inhibitor, EIPA. Representative staining images of α-SMA, a key myofibroblast marker, are shown in Figure 6A. Cells incubated with TGFβ displayed strong α-SMA staining, consistent with myofibroblast differentiation. However, co-incubation with EIPA led to a marked reduction in α-SMA staining, indicating the potential deprogramming of myofibroblasts toward a fibroblast-like state. Control cells cultured without TGFβ continued to display α-SMA staining. 

The quantification of α-SMA staining from three independent trials is presented in Figure 6B. Cells cultured with TGFβ alone showed a high percentage (90.4% ± 0.57) of moderate-to-strong α-SMA staining, confirming sustained myofibroblast differentiation. In contrast, co-incubation with EIPA significantly decreased the proportion of cells displaying moderate-to-strong staining to 13% ± 1.6% (**** *p* < 0.001), with a corresponding increase in cells exhibiting minimal α-SMA staining. These findings suggest that the inhibition of NHE1 by EIPA disrupts TGFβ-driven myofibroblast maintenance, potentially promoting de-differentiation.

While this study highlights a novel approach to attenuate myofibroblast activation and suggests the possibility of reversing differentiation, further investigations are necessary to delineate the mechanisms involved and to assess the therapeutic potential of this strategy in vivo.

## 4. Discussion

NHE1 plays a possible role in fibrosis and is a central regulator in fibroblast function. The activation of NHE1 is driven by phosphorylation and interaction with small molecules driven by signaling pathways of agonists including TGFβ, LPA, and 5HT, leading to enhanced NHE1-mediated ion transport, resulting in intracellular alkalization and extracellular acidification, which are processes required for fibroblast activation. This study investigates the involvement of NHE1 in fibrotic processes and highlights its role in disease progression.

NHE1-driven pH regulation is essential for fibroblast activation. Our results indicate significant pHi elevation in CCL39 fibroblasts treated with profibrotic agonists: TGFβ (pHi 7.52 ± 0.03), 5HT (pHi 7.47 ± 0.029), and LPA (pHi 7.63 ± 0.03). Pretreatment with EIPA, an NHE inhibitor, reduced pHi, confirming NHE1 activity and its role in these pH changes. This intracellular alkalization coupled with extracellular acidification contributes to the degradation of the ECM through processes like activating matrix metalloproteinases (MMPs), such as MMP-9 and MMP-2. These MMPs cleave the latency-associated peptide of TGFβ, thereby releasing its active form and enhancing its profibrotic signaling [30]. Consistently with previous studies showing that agonists like PE increase MMP-9 gelatinolytic activity and cell invasion in an NHE1-dependent manner [31], our results confirm that NHE1 inhibition significantly reduces TGFβ secretion. This highlights that TGFβ activation is dependent on NHE1-mediated processes. NHE1’s activity in increased extracellular acidity perpetuates TGFβ activation and fibrosis progression, creating a positive feedback loop. However, NHE inhibition effectively disrupts this loop, highlighting NHE1’s role in sustaining this fibrotic signaling.

One of the early hallmarks of fibrosis is intracellular mechanical stiffness, which is driven by cytoskeletal remodeling within the ECM. Our results demonstrate robust stress fiber formation in cells treated with TGFβ (53% ± 3.1%), 5HT (57% ± 6.3%), and LPA (63% ± 4.2%). Upon NHE1 inhibition, stress fiber formation is abolished across all tested agonists. Additionally, TGFβ, LPA, and 5HT collectively promote α-SMA expression, a hallmark of myofibroblast differentiation. These agonists induce α-SMA expression in nearly all treated cells, with varying degrees of intensity: TGFβ induced α-SMA expression in 99% of cells (80% moderate and 19% strong), LPA with 100% expression (67.2% strong), and 5HT with 100% expression (36.7% strong). With EIPA treatment, α-SMA expression significantly reduced to undetectable levels for all agonists, further confirming NHE1’s role in mediating this transition. These findings align with prior studies that highlight the significance of cytoskeletal dynamics in fibrosis, and underscore NHE1’s critical role in mechanotransduction in facilitating ECM remodeling and promoting fibroblast-to-myofibroblast differentiation.

Furthermore, chronic TGF-b exposure induces myofibroblast differentiation, characterized by elevated pHi (7.73 ± 0.026) and sustained α-SMA expression. Treatment with EIPA partially restored pHi (7.32 ± 0.072) and reduced α-SMA expression, indicating NHE1’s central role in sustaining the myofibroblast phenotype. These findings show that NHE1 inhibition can reverse this process, suggesting the possibility of myofibroblast deprogramming.

Synergistic cytokine signaling is another aspect of NHE1’s role in fibrosis. Our data revealed increased IL-6 and IL-8 secretion in response to TGFβ, 5HT, LPA, and TNFα stimulation. In most conditions examined here, EIPA treatment significantly reduced cytokine secretion, reinforcing NHE1’s role in profibrotic signaling networks.

**Figure 7:**
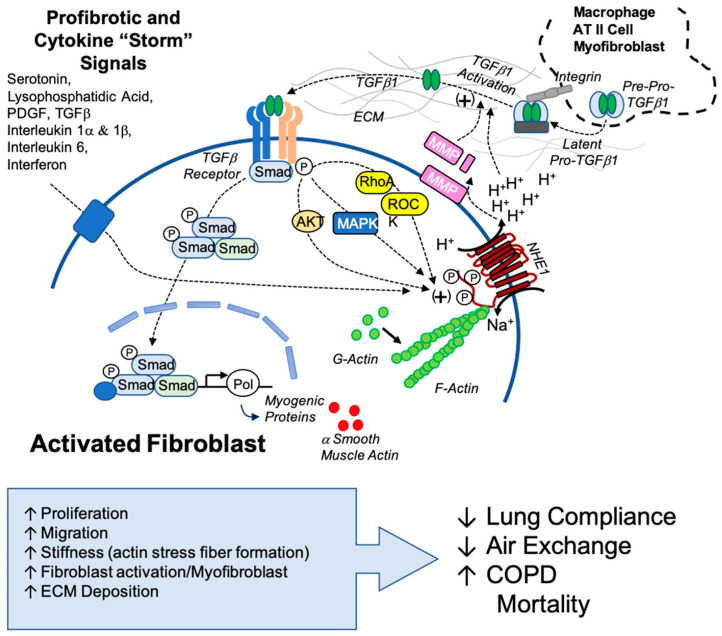
Proposed model of NHE1’s role in TGFβ1 activation and sustained profibrotic signaling. This model illustrates the interplay between NHE1 activity and TGFβ1 signaling in the fibrotic microenvironment. TGFβ1, originating from myofibroblasts, other cellular sources, or latent TGFβ1 sequestered in the extracellular matrix (ECM), is activated through ECM acidification mediated by NHE1. Acidification promotes the release of latent TGFβ1 and enhances the activity of matrix metalloproteinases (MMPs), further facilitating TGFβ1 activation. Active TGFβ1 engages its receptor, triggering downstream signaling pathways involving AKT, MAPK, RhoA, and ROCK kinase, all of which are positive regulators of NHE1. Secondary profibrotic signals, including cytokines associated with conditions like the COVID-19 cytokine storm, amplify NHE1 activity, creating a feedback loop that perpetuates ECM remodeling, myofibroblast activation, and fibrosis progression. This model highlights the central role of NHE1 in integrating profibrotic signals and sustaining the fibrotic cascade, emphasizing its potential as a therapeutic target.

Our findings highlight the novel and potentially critical role that NHE1 plays in fibrotic mechanisms and identify NHE1 as a possible therapeutic target for combatting IPF. When targeting NHE1, multiple fibrotic pathways were disrupted, including pH regulation, stress fiber formation, cytokine secretion, and myofibroblast differentiation. Not only can NHE1 be a target for lung fibrosis, but there are also implications of NHE1 being a target in similarly afflicted diseases, like SARS-CoV-2 (COVID-19). Fibrosis and severe COVID-19 share overlapping cytokine profiles, including elevated TGFβ and IL-6 levels, which suggest shared mechanisms. Our findings suggest that NHE1-mediated pH regulation and cytokine signaling may exacerbate COVID-19-associated fibrosis, providing insights into potential interventions (Figure 7).

In this study, we provide initial evidence of NHE1 as a novel and potential regulator in mechanisms that integrate profibrotic signaling through pH regulation, cytoskeletal remodeling, and cytokine networks. Future studies should focus on validating the NHE1-mediated processes in in vivo models of IPF. Particular attention should be given to understanding how NHE1-mediated pHe changes contribute to TGFβ activation through the release of latent TGFβ, the activation of MMPs, and enhanced ECM protein secretion and remodeling. Exploring NHE1 and its interaction with other ion transporters and signaling pathways will also provide a comprehensive understanding of NHE1’s involvement in fibrosis and related diseases.

## Data Availability

The original contributions presented in this study are included in the article.
